# Do Patients’ Privacy Concerns Influence Their Intention toward Medical Image Exchange Consent in Taiwan?

**DOI:** 10.3390/healthcare8010014

**Published:** 2020-01-08

**Authors:** Hsiao-Ting Tseng, Won-Fu Hung, Hsin-Ginn Hwang, I-Chiu Chang

**Affiliations:** 1 Department of Information Management, National United University, Miaoli 36003, Taiwan; appleapple928@gmail.com; 2 Department of Applied Digital Media, WuFeng University, Chiayi County 62153, Taiwan; 3 Institute of Information Management, National Chiao Tung University, Hsinchu city 30010, Taiwan; hghmis@gmail.com; 4 Department of Information Management, National Chung Cheng University, Chiayi County 62153, Taiwan

**Keywords:** privacy concerns, collection, trust, usage intention, Confucianism

## Abstract

The primary purpose of this study was to examine patients’ concerns surrounding information privacy and their intention toward medical image exchange consent. Patients’ concerns about information privacy in terms of collection, unauthorized access, errors and secondary use all have significant relationships with patients’ intention toward medical image exchange consent in Taiwan. Trust is the foundation for both parties. In this study, we aimed to determine the moderating effect of trust in order to examine patients’ intention toward medical image exchange consent under the influence of their information privacy concerns. Three hundred and fifty patients responded to the survey, which yielded a 92.3% response rate. The results of data analysis revealed that patients’ information privacy concerns had no significant relationship with patients’ intention toward medical image exchange consent. After considering the moderating effect of trust, patients’ information privacy concerns do have a significant relationship with patients’ intention toward medical image exchange consent, however, the R-square was only 4.5%. Based on this research result, we modified the research framework in order to examine patients’ information privacy concerns in terms of collection/non-collection. The R-square of the modified framework was 18.6%, and both collection and non-collection had significant relationships with patients’ intention toward medical image exchange consent. Finally, the implications, limitations and future research have been discussed.

## 1. Introduction

Protecting the privacy of patient information is always the major concern in the development of Health Information Exchange (HIE) [1,2,3]. There is no specific law or concept developed in Taiwan, and there is no successful experience of addressing patient privacy concerns in the context of HIE. The Medical Image Exchange Center (MIEC) is a typical HIE organization providing a national-level public service [4]. Therefore, issues about patient privacy protection are receiving serious attention. Dimitropoulos and Lizk [5] analyzed the United States’ policy framework that monitors and regulates HIE-related issues. Many countries have established Regional Health Information Organizations (RHIOs), and HIE is one of their activities [6,7,8,9,10], one example being the Indianapolis Network for Primary Care in central Indiana and Memphis, Western Tennessee, USA and the Electronic Child Network (eChin) in Canada. 

However, many people believe that HIE should be regulated at the national rather than regional level. According to McGraw et al. [11], based on the Health Insurance Portability and Accountability Act (HIPAA), some national-level HIE operations could develop more stringent systems to ensure patient privacy and control access to patients’ medical records [12,13,14,15,16]. Opponents believe that overly strict specifications might reduce a participant’s willingness to exchange health information [2,15,17]. Maybe one approach could be to just focus on the data not yet covered by HIPAA and then include them in HIPAA [18]. In order to protect patient privacy, provide patients with legitimate channels to obtain their own medical images, and strike a balance between public interest and medical information privacy [15], the Department of Health of Executive Yuan in Taiwan has set “the guidelines on the protection of privacy and medical image security” to ensure that medical personnel can access images only after obtaining patients’ consent and to ensure that medical institutions use an appropriate method of collecting and processing medical images. The overall purpose should be to promote public interest and provide safe and efficient medical services with legitimate use of medical images. 

The Department of Health of Executive Yuan in Taiwan has promoted MIEC. In the future, medical image exchange will provide patients with quick, convenient and high-quality medical images electronically. It will allow physicians to obtain timely and high-quality reports to help diagnosis and treatment, thereby enhancing the standard of medical care and protection of patient safety. No patient, whether seeking outpatient treatment, emergency, hospital or referral, will need to bring his/her medical images or reports (such as Computed Tomography, Magnetic Resonance Imaging and Positron Emission Tomography). This will also avoid unnecessary duplication of tests and other medical examinations.

Smith et al. [19] developed and validated an instrument that identified and measured the primary dimension of individual’s concerns about organizational information privacy practices. In a review of previous studies [19,20,21,22,23], four constructs of concern for information privacy (CFIP)’s significant situation differ from other settings. Therefore, we estimated that between these four constructs, other moderator variables may exist. Milne and Boza [24] indicated that building trust was a key element in reducing consumer privacy concerns and improving relationships between consumers and direct marketing organizations. The applications provided to patients have been implemented in advance, but the speed of the law cannot keep up with the implementation of new measures, yet the new measures have a need for standardization. In such an environment with imminent legislative action, the healthcare marketers’ ability to build and maintain consumer trust is an important step toward reducing consumer concerns and perceptions of risk. Consumers’ sensitivity towards personal information being collected and used by direct marketing firms, as well as their trust in these firms, may well determine the level of consumer concern, and ultimately, the efficacy of future healthcare delivery. It is important for policymakers to understand consumer attitudes towards personal medical information being used in direct marketing efforts [2,24,25]. Therefore, in this study, we employed “trust” as a moderator to examine concern for information privacy (CFIP) in the MIEC setting. 

Most papers that have discussed CFIP have done so in relation to Western countries (e.g., the U.S.) and in e-commerce, and it is rare to find discussions about CFIP relating to Taiwanese people and in the healthcare field. Taiwanese people, due to the effects of Confucianism, always follow orders from professionals. In the MIEC setting, these professionals are physicians. Due to Confucianism, Taiwanese people may follow physicians’ orders. Additionally, Taiwanese people often like to inquire about other people’s privacy. Therefore, in this study, we wanted to examine whether Taiwanese people’s concerns for information privacy in the MIEC setting would match with previous studies or not.

## 2. Methods

### 2.1. Methodology of the Study

This study adopted a quantitative data collection approach to empirically verify the CFIP model proposed by Smith et al. [19] in the MIEC environment, and to establish a new model in response to medical imaging situations and cultural changes.

The methodology of the research was a survey. The reasons for using a survey were as follows:

1. The proposed framework was based on prior research studies, most of which utilized a survey design. Furthermore, in this research, we wanted to examine if the model was suitable in the MIEC setting or not. The results of this study, whether they supported previous research or not, would contribute to cumulative knowledge in this field.
2. Existing instruments from previous research studies could be adopted.
3. The target population is not easy to control. As the dependent variable (intention to sign MIEC consents) varies from person to person, the analysis unit of this study was the individual.

### 2.2. Research Framework and Hypotheses

**Figure 1:**
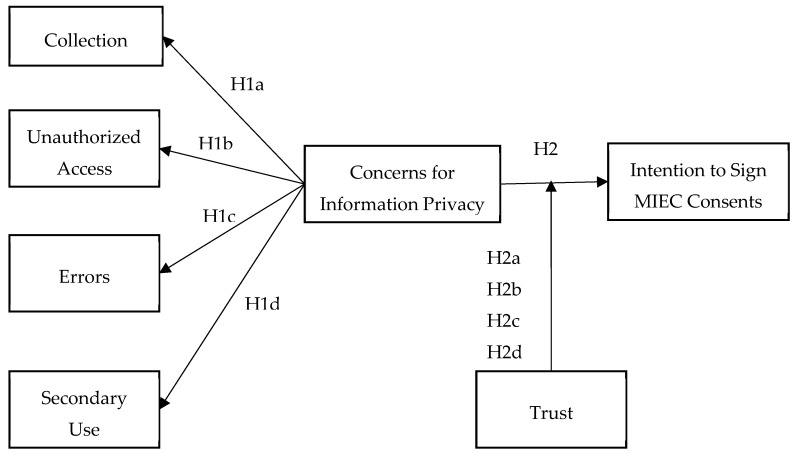
Research framework.

According to prior empirical results [23,26,27,28,29], there were four constructs of concern for information privacy that were believed to have a negative association with patients’ intention to sign MIEC consents. Then, we also used this model to examine the relationship between CFIP and intention to sign MIEC consents, which was moderated by trust. Based on the background of this research, and integrating characteristics of the medical industry in this study, the framework of this research is shown in Figure 1.

The objectives of the experimental study were to test the impact of patients’ information privacy concerns on patients’ intention to sign MIEC consents (H1, H1a, H1b, H1c, H1d) and also to test the moderating role of trust in the relationship between patients’ information privacy concerns and patients’ intention to sign MIEC consents (H2, H2a, H2b, H2c, H2d).

**Hypothesis** **1** **(H1).** *Patients’ information privacy concerns will have a negative effect on patients’ intention to sign MIEC consents.*

**Hypothesis** **1a** **(H1a).** *Patients’ information privacy concerns about collection of information by medical staff will have a negative effect on patients’ intention to sign MIEC consents.*

**Hypothesis** **1b** **(H1b).** *Patients’ information privacy concerns about unauthorized access to information by medical staff will have a negative effect on patients’ intention to sign MIEC consents.*

**Hypothesis** **1c** **(H1c).** *Patients’ information privacy concerns about errors in medical images will have a negative effect on patients’ intention to sign MIEC consents.*

**Hypothesis** **1d** **(H1d).** *Patients’ information privacy concerns about secondary use of information by medical staff will have a negative effect on patients’ intention to sign MIEC consents.*

The propensity to trust is a human personality trait that moderates the effect of trustworthiness attributes on the formation of trust [30]. This moderation effect acts positively in the sense that the higher the level of trust propensity, the greater the impact of trust attributes on the formation of trust. Some possible hypotheses for this group of factors were:

**Hypothesis** **2** **(H2).** *Patients’ trust in medical staff will moderate the effect of patients’ information privacy concerns on patients’ intention to sign MIEC consents.*

**Hypothesis** **2a** **(H2a).** *Patients’ trust in medical staff will moderate the effect of patients’ information privacy concerns about medical staff’s information collection on patients’ intention to sign MIEC consents.*

**Hypothesis** **2b** **(H2b).** *Patients’ trust in medical staff will moderate the effect of patients’ information privacy concerns about medical staff’s unauthorized access to information on patients’ intention to sign MIEC consents.*

**Hypothesis** **2c** **(H2c).** *Patients’ trust in medical staff will moderate the effect of patients’ information privacy concerns about medical image errors on patients’ intention to sign MIEC consents.*

**Hypothesis** **2d** **(H2d).** *Patients’ trust in medical staff will moderate the effect of patients’ information privacy concerns about medical staff’s secondary use of information on patients’ intention to sign MIEC consents.*

### 2.3. Questionnaire Design

The survey instrument consisted of a two-part questionnaire, as shown in Appendix A. The first part was used to collect basic information on the participating patients (Table A1). The second part of the questionnaire was based on the research by Smith et al. [19] and Gefen [31], and a 7-point Likert scale (1 indicating the most positive responses and 7 indicating the most negative responses), which was used to evaluate the patients’ information privacy concerns, trust, and intention to sign MIEC consents (Table A2).

Collection concerns center around patients’ perceptions as to whether personally identifiable medical images are being collected and stored in the MIEC appropriately. Unauthorized access reflects patients’ concerns regarding whether their medical images are available to people not properly authorized to view or work with these medical images. Concerns regarding errors relate to patients’ concerns about transmission distortion of medical images. Secondary use pertains to concerns that medical images are collected from patients for one purpose but used for another secondary purpose without authorization from the patients. 

Then, three experts in the medical industry were invited to discuss the questionnaire contents. The next step was to invite 30 patients to conduct a pilot test to ensure the clarity and objectivity of the questionnaire content in this study.

### 2.4. Sampling

The number of samples selected depended on the number of questions. The main questions in this study consisted of 21 questions. In the past, scholars suggested that the number of samples should be 5 to 10 times the number of questions. For this study, it was calculated at 10 times; therefore, at least 210 questionnaires should be collected. 

In this study, systematic sampling was used. This study conducted a questionnaire survey in Taiwanese urban hospitals between July and August 2018 and targeted outpatients as our research object. We limited our sampling frame to those who had expressed interest or were active volunteers in medical image exchange consent. During the process of collecting questionnaires, the topic attracted many interested patients to answer voluntarily, so more than the expected number of responses was collected. Among them, three hundred and fifty patients responded to the survey, which yielded a 92.3% response rate. In this study, systematic sampling was used. 

## 3. Results

A self-administered survey was used to collect data. Three hundred and fifty patients responded to the survey, which yielded a 92.3% response rate. As a result, 323 responses were retained for the subsequent analyses. Demographic data of the respondents are shown in Table 1.

Most respondents were in the age group of 20–39 years old (65.4%). Most of them were university/college students (36.8%) or masters or above (33.1%). Most of the participating patients had medical image experience (60.1%), and 59.1% of participating patients did not know about the medical image exchange center (MIEC) before we introduced MIEC to them, as shown in Table 1. In addition, up to 90.4% of participating patients had never signed consents for MIEC. 

The results of Table 1 also showed that patients’ intention to sign MIEC consent had significant differences by their age. Patients aged 60 to 69 had higher intention than patients aged 30 to 39. Moreover, patients’ concerns about collection, unauthorized access, errors, and secondary use, their trust, and their intention to sign MIEC consent had significant differences by their education. Patients with junior high education or below had higher collection concerns than patients with university education or Masters or above. Patients with senior high education had higher concerns about unauthorized access and errors than patients with junior high or below or Masters or above education. Patients with senior high education had higher secondary use concerns than patients with Masters or above. Junior high or below and senior high patients had higher trust than Masters or above patients. Senior high patients had higher intention to sign MIEC consent than junior high or below patients. 

Otherwise, considering medical imaging experience and understanding, patients with no imaging experience had higher concerns about unauthorized access, errors and secondary use than patients who had imaging experience. However, patients with imaging experience had higher intention to sign MIEC consent than patients with no imaging experience. Patients who had ever signed MIEC consents had higher secondary use concerns than patients who had never signed MIEC consents, and patients who had never signed MIEC consents had higher collection concerns than patients who had ever signed MIEC consents.

Descriptive statistics on CFIP and its four dimensions are shown in Table 2. On average, concerns about collection (mean: 3.91) were significantly higher than concerns about unauthorized access (mean: 2.44), errors (mean: 2.41) and secondary use (mean: 2.21).

### 3.1. Measurement Model Assessment

The variance inflation factor (VIF) and tolerance value were examined to detect multicollinearity. In this research, the tolerance values were 0.922, 0.336, 0.323 and 0.368, and the VIF values were 1.084, 2.973, 3.098 and 2.714. Tolerance values that were greater than 0.10 and VIF values not exceeding 10 indicated that problems of high multicollinearity were not present [32]. Therefore, in this research, the multicollinearity problem was not considered.

### 3.2. Reliability and Validity Analysis

Reliability has to do with the accuracy and precision of a measurement procedure [33,34]. Reliability is a necessary contributor to validity but is not a sufficient condition for validity. Reliability is the consistency of a set of measurements or measuring instruments. In this research, we used Cronbach’s α as the indicator to measure reliability; Cronbach’s α should be more than 0.6 in ideal conditions [35]. The reliability analysis of this research is shown in Table 3.

As shown in Table 3, the Cronbach’s α of each construct in this research was more than 0.6. Four factors of CFIP were more than 0.8, trust has the lowest reliability (0.730), and intention to sign MIEC consents had the highest reliability (0.909). Therefore, the reliability in this research was acceptable. 

Construct validity is used to identify the underlying constructs being measured and to determine how well the tests represent them [33,34]. This research used factor analysis to measure the construct validity of instruments, and used Kaiser–Meyer–Olkin (KMO) and Bartlett tests and principal components analysis. The statistics of the KMO and Bartlett tests are shown in Table 4.

The KMO measure of sampling adequacy of this research was 0.912. According to Kaiser and Rice in 1974, a Kaiser–Meyer–Olkin measure of 0.912 is excellent. The *p*-value in the Bartlett test was less than 0.001, which was a significant result. This proved that the data were worthy of processing by factor analysis.

As shown in Table 5, the factors of CFIP in this research were divided into two groups: one was collection and the other was non-collection (unauthorized access, errors and secondary use). Therefore, we modified the CFIP into two variables: collection and non-collection (including unauthorized access, errors and secondary use).

### 3.3. Testing of Hypotheses

The results of the multiple linear regressions and Moderator Regression Analysis (MRA) are shown in Table 6. 

To determine the type of moderator, the significance levels of beta coefficients were examined. If the beta coefficient on the cross-product term over the moderator is significant (sig. < 0.05), the variable is determined to be a pure moderator. If both the beta coefficients on the moderator itself and the cross-product term over the moderator are significant (sig. < 0.05), the variable is determined to be a quasi-moderator. After examination, trust was found to be a pure moderator.

H1a, H1b, H1c, and H1d were supported by the data. Hypotheses 2, 2a, 2b, 2c, and 2d discuss the relationship after moderation by trust. H2, H2a, H2b, H2c, and H2d were supported by the data.

**Figure 2:**
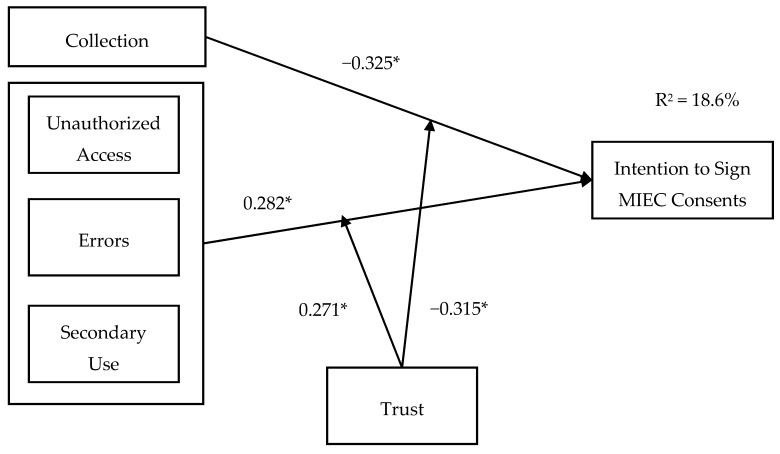
Modified research model. * *p* < 0.05.

As we found out in the factor analysis and hypotheses tests, concerns about unauthorized access, errors and secondary use were three factors that were not easy for patients to identify among each other. The reasons for this is discussed later. Therefore, we followed this research finding to refine the research model to fit patients’ information privacy concerns in the MIEC setting (as shown in Figure 2).

After refining the research model, the R^2^ changed from 0.045 to 0.186, and the conclusions and reasons for this have been discussed later.

## 4. Conclusions 

This section presents the conclusions derived from data analysis. This research examined patients’ information privacy concerns surrounding collection, unauthorized access, errors and secondary usage when their medical image information was stored in the MIEC. Therefore, we used the theory of CFIP [19] as the theoretical foundation to examine patients’ information privacy concerns about medical image exchange, and to discuss whether patients’ privacy concerns affected patients’ intention to sign MIEC consents. 

Based on the literature review, most research on CFIP was discussed in the context of e-commerce and other fields. In most of the related research, four constructs (collection, unauthorized access, errors and secondary usage) simultaneously existed in the studied settings. However, in Rose [22], the four constructs of CFIP did not co-exist. Therefore, in this research, we examined CFIP in the MIEC setting and discussed whether CFIP affected patients’ intention to sign MIEC consents. In previous research, CFIP was discussed within contexts of Western and developed countries where information privacy concerns are high and can be clearly distinguished. However, in this research, as seen from the factor analysis in Table 5, we found that the sample subjects in this study could not clearly distinguish collection and the other three constructs. Therefore, we modified CFIP into two variables: collection and non-collection. Before the research model was modified, the R^2^ was 0.045, however, after being modified, the R^2^ changed to 0.186. It was found that the low R^2^ was explained by patients’ trust in physicians and in government, and cost and convenience. Due to reasons deduced from interviews, we can see that there are still other important factors that influence patients’ intention to sign MIEC consents, such as cost and convenience. These factors had more direct influence than information privacy concerns. When we interviewed our research subjects, most showed their attitude toward privacy as if it did not matter. Most of them knew that privacy was important, but compared with information privacy concerns, loyalty to physicians/hospitals, trust in physicians/hospitals/governments and free use were the more powerful incentives for them. Under such incentives, they were willing to give up privacy. However, in the literature review, it is noted that people in developed countries have the ability to care about their health status, and in such countries, under good self-control ability, they know the importance of their privacy protection. However, the research subjects in this study were Taiwanese, and the Taiwanese culture is based on Confucianism. Education in Taiwan does not focus on issues about privacy because the Taiwanese believe in Confucianism, and Confucianism is contrary to the concept of privacy. A common saying is: “see no evil, hear no evil, speak no evil and act no evil.” This corresponds to Confucius’ ideas. The boundary line of privacy is “no evil”, and it is a fuzzy principle for our Confucianism to follow [36]. Therefore, people have a fuzzy view of the importance of privacy. However, because of the chaos of privacy, it does not affect people’s behavioral intentions. Therefore, this matched the results of this research that the Taiwanese have high information privacy concerns but are still willing to use this free, convenient, government-sponsored facility, i.e., MIEC.

Patients do have information privacy concerns about their medical images. The proposed research model was based on the assumption that patients’ privacy concerns about their medical information affect their intention to sign MIEC consents. Through our results, we found that patients’ information privacy concerns about collection, unauthorized access, errors and secondary use significantly affect patients’ intention to sign MIEC consents. We also found that patients were able to clearly distinguish collection from the other three constructs of CFIP, but they usually considered unauthorized access, errors and secondary usage together.

Data analysis showed that the relationship between patients’ information privacy concerns and patients’ intention to sign MIEC consents was significant. It showed that patients do have information privacy concerns and these concerns really do affect their intention to sign MIEC consents. The results also showed that patients have information privacy concerns about collection and they cannot distinguish between unauthorized access, errors and secondary usage. The relationship between CFIP and intention to sign MIEC consents was significant. If we added trust to moderate this relationship, it would be more significant than in the original model. Trust in this model played the role of a complete moderator. In other words, patients’ trust in physicians and the government was a key factor that affected their intention to sign MIEC consents. Through interviews, we found that there were four main reasons, which were as follows. MIEC is free for users when they need it in the future, MIEC is a convenient choice for them, patients trust physicians’ requests, and patients trust government policy. This shows that patients’ reasons derived from interviews fit the data analysis results of this research.

## 5. Recommendations

Szase and Hollender [37] proposed three kinds of physician–patient interaction models: the activity-passivity model, the guidance-cooperation/paternalistic model and the mutual participation model. In Szase and Hollender’s opinion, the mutual participation model was the best condition. However, in Taiwanese culture, most patients stay in the activity-passivity model and guidance-cooperation/paternalistic model. Because of asymmetric information between physicians and patients, patients always rely on physicians’ opinions. Therefore, they trust physicians and follow physicians’ orders. Furthermore, they also trust government decisions and policy. Interviews showed that patients believed physicians and that governments always did well for them. Patients also believed physicians and governments protected their information privacy, and they had no doubt about it.

### 5.1. For Government 

Suggestions are offered to the government on how to educate the public to maintain and protect Electronic Medical Records (EMR) privacy, on how and when to disclose their private information, and on how to help people to really protect their EMR. It has also been shown that patients trust government decisions and policy, and therefore the government should encourage the public to use EMR more positively. Patients do have concerns about the collection, unauthorized access, errors and secondary use of their medical information; therefore, the government should strengthen policy and information security regarding patients’ medical information to keep patients’ trust.

### 5.2. For Medical Care Service Providers and Patients

This research was expected to help reduce the resistance to MIEC’s implementation caused by patients’ privacy concerns. With the success of MIEC, the costs for patients and medical institutions can be reduced to achieve a win-win situation for all. Patients believe that medical care services protect their information privacy, and therefore medical care services should maintain trust to keep the ideal physician–patient relationship.

### 5.3. For Academia

Through this research, academia can understand the impact of MIEC implementation on patients’ privacy concerns, and this research can be a reference for future researchers. This research shows that there are still other factors that affect patients’ intention to sign MIEC consents. This research shows the direction for academia to find the other factors between CFIP and intention to sign MIEC consents in the healthcare field.

### 5.4. Limitations

Like other empirical research studies, this research suffers from several limitations in relation to time and human resources. The research subjects in this research were confined to Southern Taiwan. It was not possible to contact many patients having a mutual participation model of the physician–patient relationship. Therefore, maybe it cannot fully reflect patients’ opinions about information privacy concerns and their intention to sign MIEC consents.

Cultural difference was a key factor that influenced the findings of this research and resulted in the low coefficient of determination in the statistical results. Developed countries have always had high concerns about human rights but Taiwan is a developing territory and people have long believed and lived in Confucian culture, and therefore people in our territory follow orders from medical professionals. Therefore, it is suggested that future research can continue to explore the results caused by cultural differences, which will help to understand the context of privacy under a cross-cultural influence.

## Figures and Tables

**Table 1:** Demographics for the sample population.

Variable	Description	Frequency	%	F-Value					
				C	U	E	S	T	I
Gender	Male	158	48.9	7.910	0.979	0.328	0.120	1.189	7.178
	Female	165	51.1						
Age Group	Younger than 20	15	4.6	2.780	0.731	1.520	2.522	3.234	4.830 *
	20–29	111	34.4						
	30–39	100	31.0						
	40–49	54	16.7						
	50–59	22	6.8						
	60–69	8	2.5						
	70 or older	13	4.0						
Education	Junior High or below	44	13.6	3.377 *	3.736 *	4.531 *	4.732 *	6.336 *	3.678 *
	Senior High	53	16.4						
	University/College	119	36.8						
	Masters or above	107	33.1						
Medical Image Experience	No	129	39.9	2.170	0.609 *	7.021 *	9.423 *	0.459	1.151 *
	Yes	194	60.1						
Knew of MIEC in advance	No	191	59.1	0.042	0.357	2.806	1.438	1.444	1.676
	Yes	132	40.9						
Ever signed consent for MIEC	No	292	90.4	0.827 *	0.567	0.069	0.690 *	0.553	0.954
	Yes	31	9.6						

* *p* < 0.05; C: collection; U: unauthorized access; E: errors; S: secondary use; T: trust; I: intention to sign MIEC consents.

**Table 2:** Descriptive statistics on concern for information privacy (CFIP).

Concern for Information Privacy Items (1, Strongly Agree; 4, So-So; 7, Strongly Disagree)	Mean	S.D.
Average collection	3.91	1.26
Average unauthorized access	2.44	1.23
Average errors	2.41	1.23
Average secondary use	2.21	1.22
Average CFIP	2.75	1.01

S.D.: standard deviation.

**Table 3:** Reliability analysis.

Constructs	Cronbach’s α
Collection	0.861
Unauthorized Access	0.813
Errors	0.896
Secondary Use	0.879
Trust	0.730
Intention to Sign MIEC Consents	0.909

**Table 4:** Kaiser–Meyer–Olkin (KMO) and Bartlett tests.

Factor Analysis Items		Value
Kaiser–Meyer–Olkin Measure of Sampling Adequacy		0.912
Bartlett’s Test of Sphericity	Approx. Chi-Square	3237.004
	df	105
	Sig.	0.000 *

* Sig: significance, *p* < 0.05.

**Table 5:** Component matrix and rotated component matrix.

	Component		Component	
	1	2	1	2
C_1		0.813		0.860
C_2		0.701		0.758
C_3		0.791		0.841
C_4		0.836		0.874
U_1	0.697		0.653	
U_2	0.791		0.779	
U_3	0.814		0.813	
E_1	0.758		0.779	
E_2	0.831		0.837	
E_3	0.835		0.841	
E_4	0.790		0.801	
S_1	0.750		0.769	
S_2	0.809		0.819	
S_3	0.798		0.797	
S_4	0.745		0.745	

Method: Principal components.

**Table 6:** Moderator Regression Analysis results.

Title	Relationship	Std. Beta Coefficient	t-Value (Sig. t)	R^2^ Changed (Sig F)	Total of R^2^	Hypothesis Confirmed?
H1	CFIP→I	0.074	1.322	0.005	0.005	No
H2	+Trust	0.185	3.375 * (0.001)	0.034 * (0.001)	0.040	Yes (pure Moderator)
	+Trust*CFIP	−0.250	−1.333 (0.184)	0.005 (0.184)	0.045	
H1a	C→I	−0.262	−4.871 *	0.069	0.069	Yes
H2a	+Trust	0.174	3.269 * (0.001)	0.030 * (0.001)	0.099	Yes (pure Moderator)
	+Trust*C	−0.231	−0.231 (0.181)	0.005 (0.181)	0.104	
H1b	U→I	0.157	2.856 *	0.025	0.025	Yes
H2b	+Trust	0.185	3.412 * (0.001)	0.034 * (0.001)	0.059	Yes (pure Moderator)
	+Trust*U	−0.222	−1.657 (0.099)	0.008 (0.099)	0.067	
H1c	E→I	0.233	4.289 *	0.054	0.054	Yes
H2c	+Trust	0.169	3.142 * (0.002)	0.028 * (0.002)	0.083	Yes (pure Moderator)
	+Trust*E	−0.199	−1.381 (0.168)	0.005 (0.168)	0.088	
H1d	S→I	0.178	3.234	0.032	0.032	Yes
H2d	+Trust	0.177	3.271 * (0.001)	0.031 * (0.001)	0.063	Yes (pure Moderator)
	+Trust*S	−0.257	−1.922 (0.055)	0.011 (0.055)	0.074	

* *p* < 0.05; C: collection; U: unauthorized access; E: errors; S: secondary use; T: Trust; I: Intention to Sign MIEC Consents.

## Appendix A

**Table A1:** Questionnaires: Demographic Information.

1. Gender		3. Education	
	Male		Junior high or less
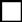
	Female		Senior high school
			College/University
			Graduate
		4. Imaging experiences	
2. Age (years)			No
	Under 20		Yes
	20–29	5. Understand MIEC	
	30–39		No
	40–49		Yes
	50–59	6. Ever Signed MIEC consent	
	60–69		No
	More than 70		Yes

In this part, the aim of this study is to understand about your opinion. Please rate the following questions on a scale 1 to 7 to describe your opinion. (1—Strongly disagree, 4—neutral, 7—strongly agree).

**Table A2:** Questionnaires: Citizens’ Information Privacy Concerns and Their Intention to Use MIEC.

Questions	1	2	3	4	5	6	7
*Collection*							
1. It usually bothers me when my images information uploads to MIEC.							
2. When medical staff ask me for personal images information in MIEC, I sometimes think twice before providing it.							
3. It bothers me to upload personal images information to MIEC.							
4. I’m concerned that medical staff are collecting too much personal images information about me.							
*Unauthorized access*							
1. Medical staff should devote more time and effort to preventing unauthorized access to personal images information.							
2. IEC that contain personal images information should be protected from unauthorized access –no matter how much it costs.							
3. Medical staff should take more steps to make sure that unauthorized people cannot access personal images information in their computers.							
*Errors*							
1. All the personal images information in MIEC should be double-checked for accuracy—no matter how much this costs.							
2. Medical staff should take more steps to make sure that the personal images information in their files is accurate.							
3. Medical staff should have better procedure to correct errors in personal images information.							
4. Medical staff should devote more time and effort to verifying the accuracy of the personal images information in MIEC.							
*Secondary Use*							
1. Medical staff should not use personal images information for any purpose unless it has been authorized by the individuals who provided the images information.							
2. When people give personal images information to medical staff for some reason, the medical staff should never use the information for any other reason.							
3. Medical staff should never sell the personal images information in MIEC to other medical staff.							
4. Medical staff should never share personal images information with other medical staff unless it has been authorized by the individuals who provided the images information.							
*Trust*							
1. I generally trust other people.							
2. I generally have faith in humanity.							
3. I feel that people are generally reliable.							
4. I tend to count upon other people.							
*Intention to use MIEC*							
1. I intend to use MIEC at every opportunity over the next year.							
2. I plan to increase my use of MIEC over the next year.							
